# A case report of small intestinal volvulus caused by lipomatosis of the small intestine successfully treated with enterectomy

**DOI:** 10.3389/fonc.2024.1415211

**Published:** 2024-06-28

**Authors:** Hao Qin, Yu Wei Zhao, Xiao Zhou Wang, Lei Jiang, Qiang Liu, Zhan Wu Li, Guang Sheng Zhao

**Affiliations:** ^1^ Department of Acute abdominal surgery, The Second Hospital of Dalian Medical University, Dalian, Liaoning, China; ^2^ Department of Oncology, Affiliated Zhongshan Hospital of Dalian Universtity, Dalian, Liaoning, China; ^3^ Department of Emergency surgery, Affiliated Zhongshan Hospital of Dalian Universtity, Dalian, Liaoning, China

**Keywords:** small intestinal tumor, lipomatosis, intestinal volvulus, small intestine, resection

## Abstract

Small intestinal lipomatosis is a rare condition that presents a diagnostic challenge due to the absence of identifiable clinical symptoms and limitations of small intestine examination methods. Consequently, preoperative diagnosis is difficult and only a limited number of cases have been documented in the scientific literature. Here, we report a rare case of volvulus caused by small intestinal lipomatosis. A 58-year-old female patient was tentatively diagnosed with acute ileus. The whirl sign was detected using abdominal three-dimensional enhanced computed tomography, along with marked local intestinal dilation and multiple irregular fat-like containing lesions. During surgery, abnormal dilation of the small intestine between 80 and 220 cm from the ileocecal valve was detected and the affected intestine displayed a folded and twisted configuration. Examination of the resected intestine showed that the inner wall of the diseased intestinal lumen was covered with more than 100 lipomas of different sizes, the largest of which measured ~8.0 cm in diameter. Based on clinical symptoms alone, it was difficult to identify the cause of intestinal volvulus before surgery. Complete resection of the affected small intestine and subsequent pathological analysis yielded a definitive diagnosis of small intestinal lipomatosis. While small intestinal lipomatosis is a rare condition, prognosis is favorable if diagnosed early and treated appropriately. The application of three-dimensional enhanced computed tomography imaging can aid in accurate diagnosis, while complete resection of the affected small intestine is crucial to improve patient prognosis.

## Introduction

Small intestine tumors account for only 1-2% of all digestive tract tumors, among which ~30% are considered benign lesions (1). Lipomas are most commonly detected in subcutaneous tissues but can occasionally develop in the digestive tract (2). Lipomas of the small intestine are typically solitary. However, limited cases displaying large numbers have been reported, clinically referred to as small intestinal lipomatosis (3). The incidence of this rare benign tumor ranges from 0.04 to 4.5% (4). Given its asymptomatic presentation, the condition often goes undetected (5) until the tumor grows sufficiently large to cause acute abdominal symptoms and pain (6). Preoperative diagnosis is challenging due to the absence of specific clinical manifestations and ineffective small intestine examination modalities, leading to a high incidence of misdiagnosis or missed diagnosis. In this report, we describe a rare case of volvulus caused by small intestinal lipomatosis.

## Presentation of the case

A 58-year-old female patient previously diagnosed with acute ileus was transferred to our hospital on July 16, 2021. She presented with abdominal pain and distension, vomiting, and cessation of flatulence and defecation for 2 days. The patient had no known history of health issues prior to admission.

Abdominal physical examinations on admission were conducted as follows:

Inspection: protuberance of abdomen and intestinal patterns were observed.

Palpation: abdominal tenderness without rebound tenderness or muscle tonus, no palpable liver and spleen below the costal margin, and no palpable abnormal mass in the abdomen.

Percussion: percussion of whole abdomen showed tympanic resonance and negative shifting dullness.

Auscultation: bowel sounds were audible at a rate of 8 times/min as well as the sound of gas passing through water, but no vascular murmur was detected.

The body mass index (BMI) of the patient was 19.29.

**Figure 1:**
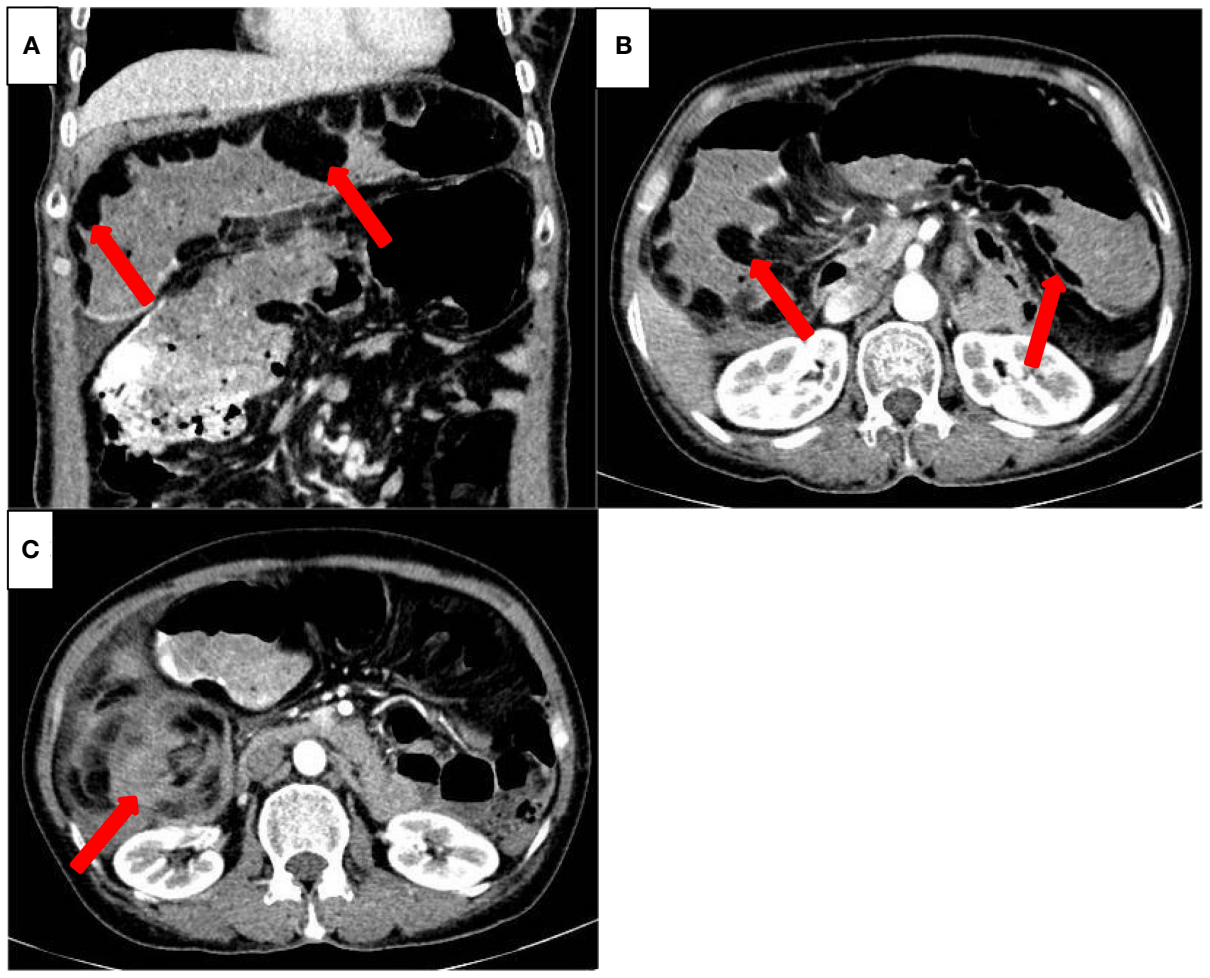
Preoperative three-dimensional enhanced abdominal CT (transverse view **(A)**, coronal view **(B)** Multiple, irregular lipoid-like density foci (indicated by red arrow) can be seen in the enlarged small intestinal cavity, that showed no enhancement on the enhanced CT scan. **(C)** The small intestine appears swirling (red arrow).

Laboratory parameters were as follows: white blood cells 12.74×10^9^/L (normal range, 3.5-9.5×10^9^/L), neutrophils 85.7% (normal range, 40-75%), hemoglobin 102 g/L (normal range, 130-175 g/L), and procalcitonin 0.15 ng/mL (normal range, 0-0.05 ng/mL). Enhanced abdominal CT revealed a swirling distribution of the small intestine in the right middle and lower abdomen, thickening and narrowing of the local intestinal wall, significant dilation of the area above the lumen stenosis, presence of gas and fluids in the cavity, and multiple irregular lipoid-like density foci in the abdominal cavity that were not enhanced (**Figures 1A–C**).

The initial diagnosis was multiple masses in the small bowel with secondary volvulus, requiring emergency exploratory laparotomy. During surgery, abnormal dilation of the small bowel was observed between 80 and 220 cm from the ileocecal valve, with a maximum diameter of ~14 cm, and the diseased intestine was folded and twisted. Subsequently, complete resection of the affected intestine was performed, followed by primary anastomosis.

**Figure 2:**
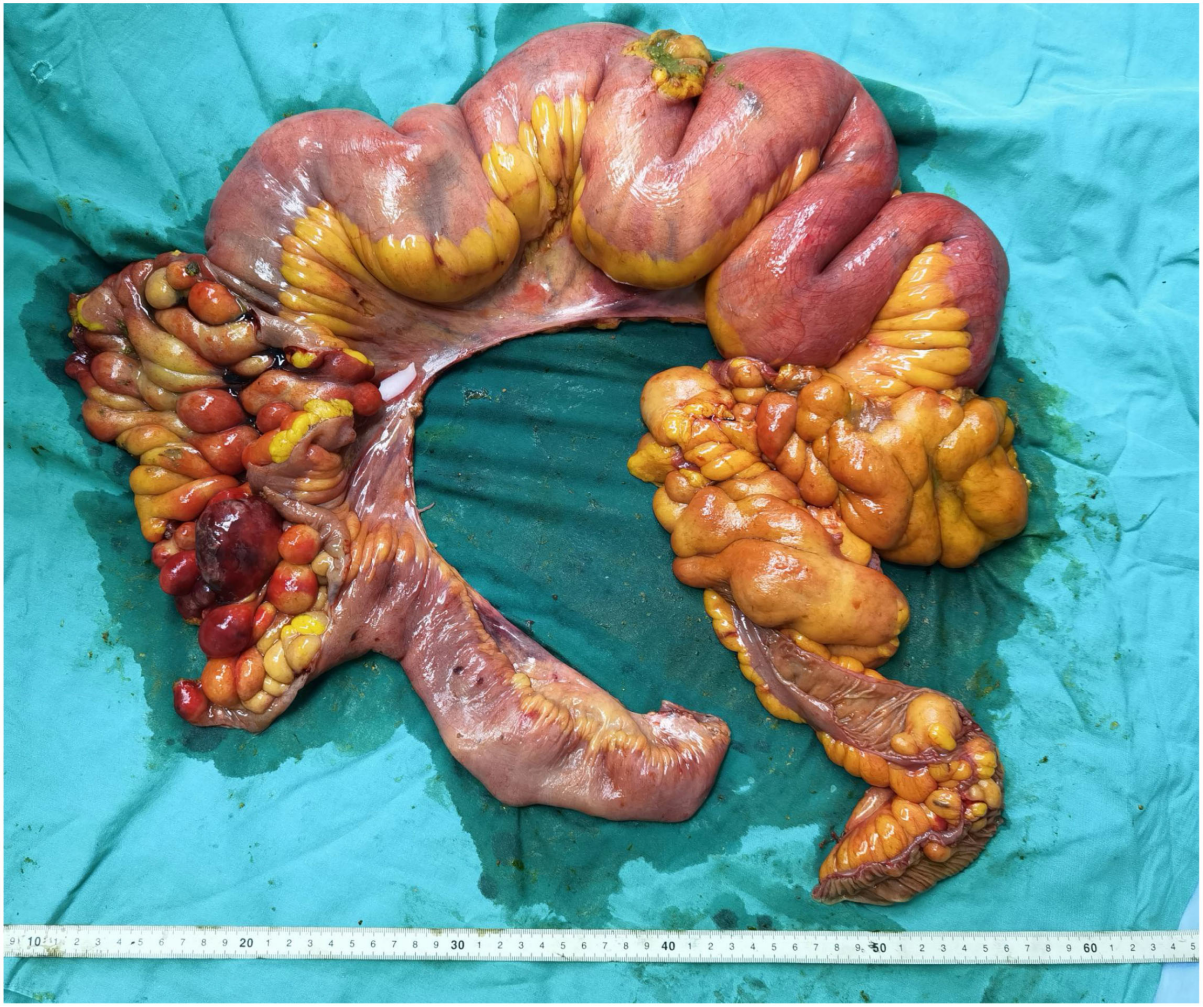
The gross specimen revealed lipomas of different sizes densely covering the inner wall of the diseased intestinal cavity.

**Figure 3:**
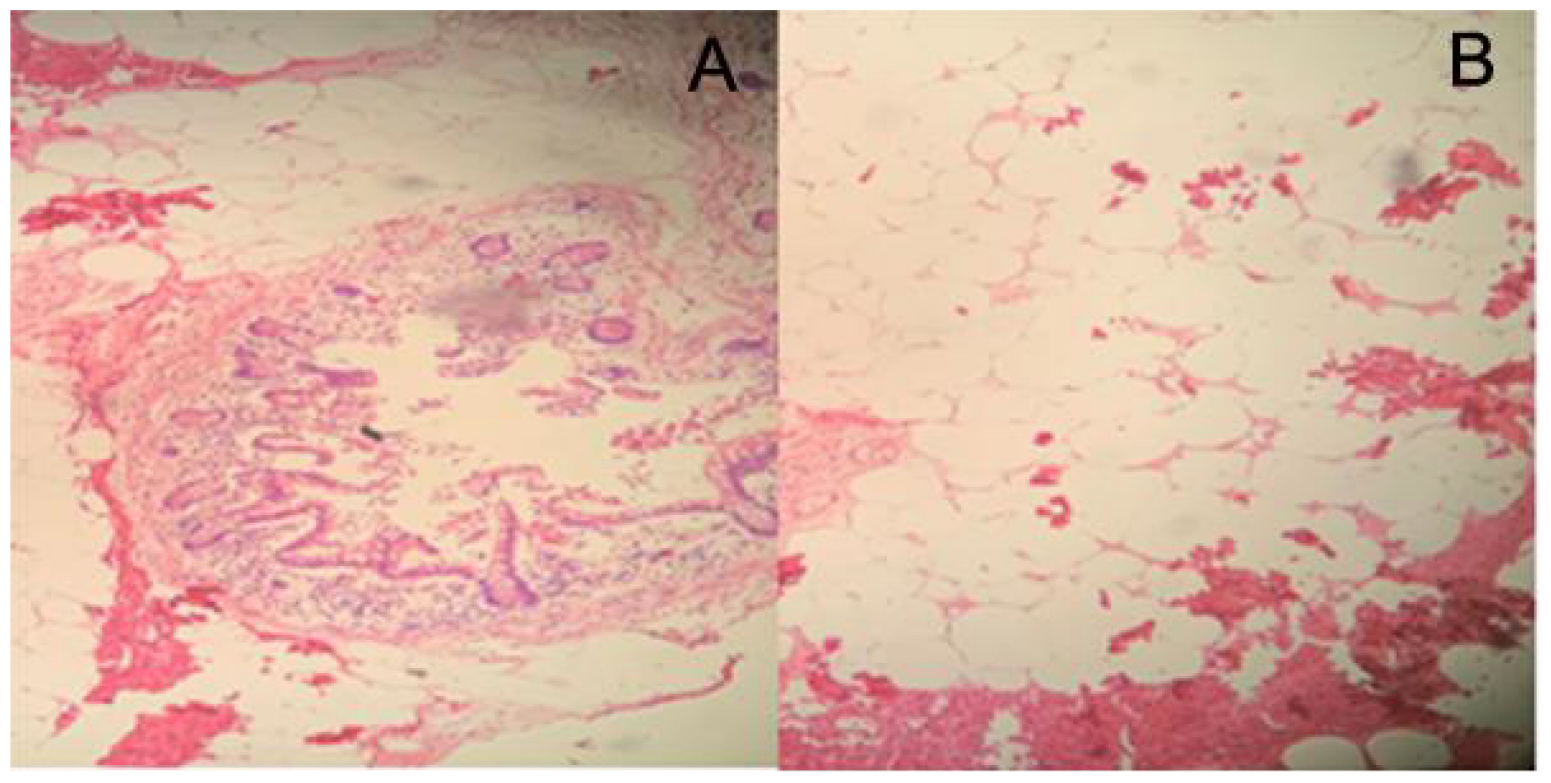
Pathological examination showed a submucosal lesion that was composed of mature adipose tissue (HE staining, **A**×50, **B**×100).

**Figure 4:**
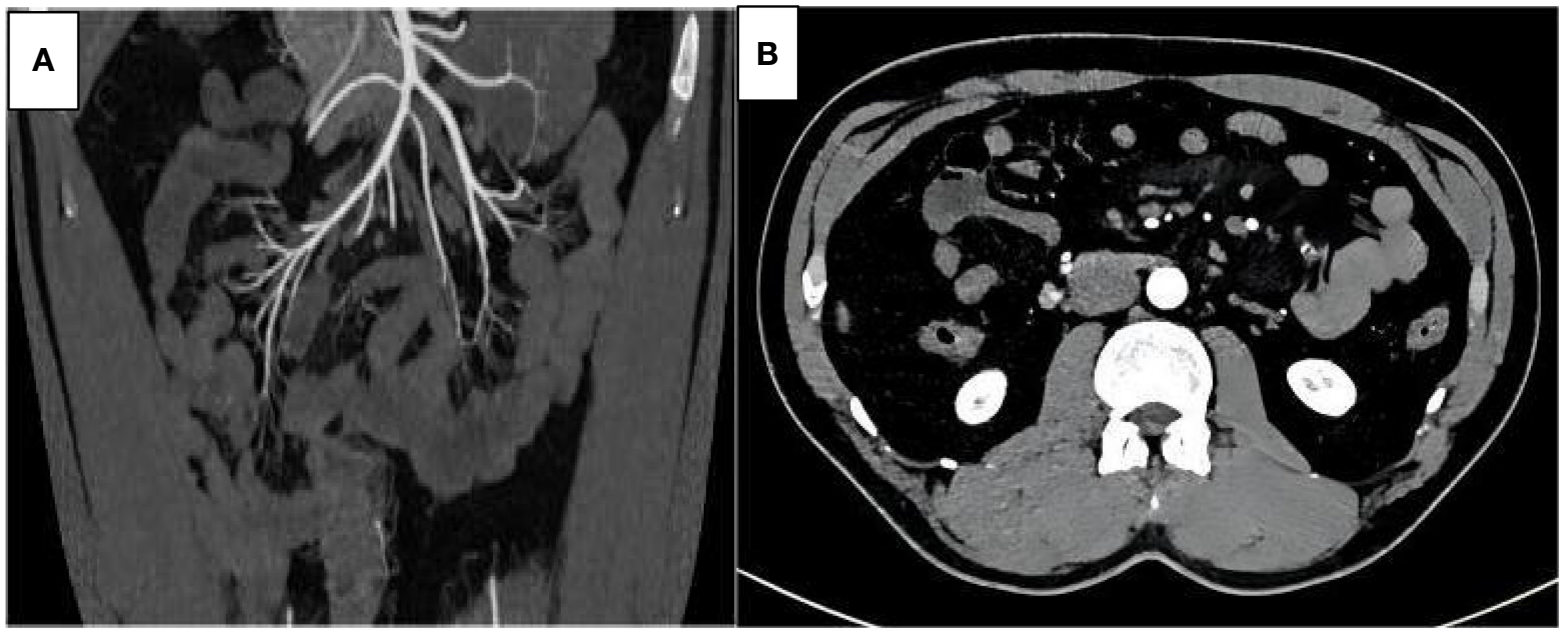
Three-dimensional enhanced abdominal CT scan (**A** transverse view, **B** coronal view) 20 months after surgery: no recurrence of small intestinal lipomatosis was observed.

Specimen examination revealed that the inner wall of the diseased intestinal lumen was covered with over 100 lipomas of varying sizes, with the largest measuring around 8.0 cm in diameter, in addition to scattered necrotic bleeding points and abnormal mesentery fat distribution (**Figure 2**). Postoperative pathological diagnosis indicated small intestinal lipomatosis with local hemorrhage (**Figures 3A, B**). The patient recovered successfully after surgery and was discharged on July 23, 2021. After 20 months of follow-up, no recurring symptoms of discomfort were reported. The BMI of the patient increased to 21.79 and abdominal enhanced CT imaging revealed no signs of recurrence (**Figures 4A, B**).

## Discussion

Small intestinal lipomatosis is a rare clinical condition characterized by diffuse and uneven excessive hyperplasia of mature adipose tissue that is deposited in the submucosa of the small intestine, forming irregular masses of varying sizes. While the pathogenesis of this disease is not yet fully understood, several factors may contribute to its development, including fatty deformation of connective tissue in the small intestine, fat infiltration around the fiber trabecular tube, dysplasia of the local fat blood supply and lymphatic circulation, and local metabolic dysfunction due to chronic inflammatory stimulation of the intestine (7–9). The patient in this report had a normal BMI and no family history of intestinal lipomatosis. Mesangial fat hyperplasia was observed in the area where the diseased intestine was located. Previous reports of cases exhibiting similar symptoms did not have corresponding family histories. Therefore, the onset of the disease may not be associated with genetic factors, environmental factors, or obesity (10).

The lesion location in this patient is consistent with small intestinal lipomatosis, which typically affects a specific section of the intestine, most commonly the ileum, according to previous reports (11). In the early stages, most patients generally exhibit no obvious clinical symptoms (12). With progressive tumor growth, symptoms, such as intestinal obstruction, intussusception, intestinal volvulus, and digestive tract bleeding, often emerge and may be secondary to the initial treatment (13). Qi et al. (14) reviewed 26 cases of intestinal lipomatosis reported in the literature, among which 20 had clinical manifestations of abdominal pain with anemia and six reported abdominal pain with vomiting. CT examination revealed intestinal wall thickening in 18 cases, intestinal diverticulum in two cases, and intestinal obstruction or intussusception in six cases. In this case, the patient had abdominal pain and vomiting accompanied by cessation of gas and defecation, which were consistent with acute intestinal obstruction. The patient additionally developed intestinal volvulus that has not been reported in previous cases.

Intestinal volvulus, one of the most serious and rapidly evolving intestinal obstruction disorders, is characterized by obstruction of both the intestinal lumen and mesenteric blood circulation (15). The primary causes of intestinal volvulus include: (1) anatomical factors, such as postoperative adhesions, congenital midgut malrotation, or lengthy intestinal canal, (2) physical factors, such as increase in the weight of the intestinal loop itself (for instance, following a large amount of food in the intestinal lumen after a full meal, intestinal tumors, or a significant volume of dry stool accumulating in the bowel), and (3) motility factors, such as strong intestinal peristalsis or a sudden change in body position. In this study, a large number of lipomas in the diseased intestine may have led to an increase in bowel weight, and during strong bowel movement or a sudden change in body position, unsynchronized movements of intestinal loops could occur, resulting in intestinal volvulus.

Based on clinical symptoms alone, it was difficult to identify the cause of intestinal volvulus prior to surgery. Three-dimensional CT of the small intestine can provide important diagnostic information by showing dilatation of the diseased small intestine, multiple different-sized, irregular low-density shadows in the cavity, and localized thickening and narrowing of the intestinal wall (2, 16). In cases where CT scans are inconclusive, other procedures, such as X-ray barium enterography, multimodal small bowel magnetic resonance imaging, double balloon enteroscopy, or small intestine capsule endoscopy, may be effectively used. These imaging findings provide a strong basis for preoperative diagnosis of the etiology. The definitive diagnosis of lipomatosis is still primarily based on histopathology, manifesting as diffuse adipose tissue infiltration in the intestinal mucosa and muscular bundle, along with microscopic lesions usually consisting of sheet-like mature adipose cells that resemble normal adipose tissue and are immunohistochemically positive for Vimentin and S-100 (14, 17).

Small intestinal lipomatosis is a benign tumor that is usually slow-growing and rarely malignant. Surgical excision is the preferred treatment option (18). In our patient, the operation revealed thickened inner wall of the diseased intestinal cavity and dense coverage with soft tissue of different sizes, some of which were accompanied by intestinal obstruction and intussusception. Notably, incomplete resection may result in recurrence due to the diffuse infiltrating growth of adipose tissue in the submucosa of the intestinal wall and between muscular layers (14). For instance, a case report by Yu et al. (19) described a recurrence 14 years after local surgical resection. Tawfik and co-workers performed surgical treatment on eight cases of small intestinal lipomatosis, five receiving complete resection of the diseased intestine without postoperative recurrence and three that underwent local resection with postoperative recurrence in all cases (20). The patient in this case report underwent complete resection of the diseased bowel and was followed up for 20 months with no signs of recurrence, indicating a significant effect of complete resection of the tumor. Due to the relatively short follow-up time, the long-term efficacy of the procedure in the patient is yet to be established.

## Conclusions

Small intestinal lipomatosis is a rare condition. Its presentation as a complicated intestinal volvulus is even rarer, although prognosis is favorable if diagnosed early and treated appropriately. However, preoperative diagnosis is challenging, often resulting in misdiagnosis or missed diagnosis. Three-dimensional enhanced CT scanning can aid in the preoperative identification of the condition, while complete resection of the affected bowel is crucial for improving patient outcomes.

## Data availability statement

The original contributions presented in the study are included in the article/supplementary material. Further inquiries can be directed to the corresponding author/s.

## Ethics statement

This study has been approved by the ethics committee of Affiliated Zhongshan Hospital of Dalian Univertsity. The studies were conducted in accordance with the local legislation and institutional requirements. The participants provided their written informed consent to participate in this study. Written informed consent was obtained from the individual(s) for the publication of any potentially identifiable images or data included in this article.

## Author contributions

QH: Formal analysis, Methodology, Writing – original draft, Writing – review & editing. YZ: Formal analysis, Methodology, Writing – original draft, Writing – review & editing. XW: Data curation, Resources, Writing – original draft, Writing – review & editing. LJ: Data curation, Resources, Writing – original draft, Writing – review & editing. QL: Data curation, Resources, Writing – original draft, Writing – review & editing. ZL: Conceptualization, Project administration, Writing – original draft, Writing – review & editing. GZ: Conceptualization, Project administration, Writing – original draft, Writing – review & editing.
